# Human papillomavirus types in cervical high‐grade lesions or cancer among Nordic women—Potential for prevention

**DOI:** 10.1002/cam4.1961

**Published:** 2019-01-11

**Authors:** Cecilie Dovey de la Cour, Sonia Guleria, Mari Nygård, Laufey Trygvadóttir, Kristjan Sigurdsson, Kai‐Li Liaw, Maria Hortlund, Camilla Lagheden, Bo T. Hansen, Christian Munk, Joakim Dillner, Susanne K. Kjaer

**Affiliations:** ^1^ Unit of Virus, Lifestyle and Genes Danish Cancer Society Research Center Copenhagen Denmark; ^2^ Department of Research Cancer Registry of Norway Oslo Norway; ^3^ Icelandic Cancer Registry Icelandic Cancer Society Reykjavik Iceland; ^4^ Faculty of Medicine University of Iceland Reykjavik Iceland; ^5^ Epidemiology Merck Kenilworth New Jersey; ^6^ Department of Laboratory Medicine Karolinska Institute Stockholm Sweden; ^7^ Department of Obstetrics and Gynecology Copenhagen University Hospital, Rigshospitalet Copenhagen Denmark

**Keywords:** cervical cancer, cervical intraepithelial neoplasia, human papillomavirus, nordic countries, prevalence

## Abstract

It is valuable to establish a population‐based prevaccination baseline distribution of human papillomavirus (HPV) types among women with high‐grade cervical intraepithelial neoplasia (CIN) grade 2 or 3 and cervical cancer in order to assess the potential impact of HPV vaccination. In four countries (Denmark, Norway, Sweden, and Iceland), we collected consecutive series of cervical cancers (n = 639) and high‐grade precancerous cervical lesions (n = 1240) during 2004‐2006 before implementation of HPV vaccination and subjected the specimens to standardized HPV genotyping. The HPV prevalence was 82.7% (95% confidence interval [CI] 79.0‐86.4) in CIN2, 91.6% (95% CI 89.7‐93.5) in CIN3, and 86.4% (95% CI 83.7‐89.1) in cervical cancer. The most common HPV types in CIN2/3 were HPV16 (CIN2: 35.9%, 95% CI 31.2‐40.6; CIN3: 50.2%, 95% CI 46.8‐53.6) and HPV31 (CIN2: 10.9%, 95% CI 7.8‐13.9; CIN3: 12.1%, 95% CI 9.9‐14.3), while HPV16 and HPV18 were the most frequent types in cervical cancer (48.8%, 95% CI 44.9‐52.7 and 15.3%, 95% CI 12.5‐18.1, respectively). The prevalence of HPV16/18 decreased with increasing age at diagnosis in both CIN2/3 and cervical cancer (*P* < 0.0001). Elimination of HPV16/18 by vaccination is predicted to prevent 42% (95% CI 37.0‐46.7) of CIN2, 57% (95% CI 53.8‐60.5) of CIN3 and 64% (95% CI 60.3‐67.7) of cervical cancer. Prevention of the five additional HPV types HPV31/33/45/52/58 would increase the protection to 68% (95% CI 63.0‐72.2) in CIN2, 85% (95% CI 82.4‐87.2) in CIN3 and 80% (95% CI 77.0‐83.2) in cervical cancer. This study provides large‐scale and representative baselines for assessing and evaluating the population‐based preventive impact of HPV vaccination.

## 1 INTRODUCTION

Globally, more than 500 000 women are diagnosed every year with cervical cancer, and around 260 000 women die, making it the fourth most common cancer in women.^1^ Normal cervical epithelium may develop through high‐grade cervical intraepithelial neoplastic (CIN) lesions into cervical cancer. This transition requires persistent infection with oncogenic types of human papillomavirus (HPV),^2^ of which HPV16 and 18 have the greatest oncogenic potential.^3^, ^4^ Together, HPV16/18 causes approximately 70% of cervical cancer.^3^, ^4^

Since licensure of the first HPV vaccine in 2006, the four countries in this study have implemented HPV vaccination in their national immunization programs. Denmark and Norway initiated HPV vaccination in 2009, followed by Iceland (2011) and Sweden (2012), with all HPV vaccination programs targeting 10‐12 years old girls. In Denmark, a catch‐up program aimed at girls aged 13‐15 years was included from 2009 and was in 2012 expanded to women up to 27 years of age. In Sweden, girls aged 13‐17 years had access to subsidized HPV vaccination during 2007‐2011, while the national vaccination program in 2012 included a catch‐up program with free vaccination of girls aged 13‐18 years.^6^ The vaccines used in the programs targeted either four (16/18 and 6/11) or two (16/18) HPV types. A recently launched HPV vaccine targets five additional oncogenic HPV types (31/33/45/52/58). The generally high participation rates (50%‐90%) in both HPV vaccination programs and cervical screening programs in the Nordic female populations,^6^ as well as the comprehensive registries and biobanking systems, make these countries an ideal setting for studying the population‐based preventive effects of HPV vaccines. The vaccination programs in Denmark and Sweden have been shown to reduce the incidence of genital warts (90% of which are caused by HPV6/11) and cervical precancerous lesions.^7^, ^8^ It will take longer before a decrease in the occurrence of cervical cancer can be observed. We wished to establish a Nordic baseline distribution (ie, prior to the vaccine initiation) of HPV types in cervical cancer and high‐grade cervical lesions as a basis for assessment and future evaluation of the population‐preventive impact of HPV vaccination and potential HPV type replacement. Previous studies have reported a higher prevalence of HPV16/18 with increasing severity of cervical lesions.^10^, ^11^ However, only few studies have examined the age‐specific distribution of HPV types among population‐based series of women with either cervical cancer or high‐grade precancerous cervical lesions.^4^, ^13^, ^14^

The aim of this study was to assess the prevalence and age‐specific distribution of HPV types in different grades of severe CIN (grades 2 and 3) and cervical cancer among women from Denmark, Iceland, Norway, and Sweden, during 2004‐2006. This will enable establishment of a baseline for surveillance of the effect of HPV vaccination as well as an estimation of the proportion of CIN2, CIN3, and cervical cancer that could potentially be prevented when women are vaccinated either against two (16/18) or seven (16/18/31/33/45/52/58) oncogenic HPV types.

## 2 MATERIALS AND METHODS

### 2.1 Study design

This is a multicountry, cross‐sectional study including four countries (Denmark, Norway, Sweden, and Iceland). We aimed to collect formalin‐fixed paraffin‐embedded cervical histological specimens of 200 cervical cancers and 300 CIN2 or CIN3, randomly selected from each country, with a smaller number of cancers from Iceland due to the limited size of the female population. Women with a histologically confirmed diagnosis of CIN2, CIN3, or cervical cancer, during 2004‐2006, were eligible for the study (2000‐2008 for women with cervical cancer in Iceland). The study was approved in each country by the relevant authorities; the Scientific Ethics Committee and the Data Protection Board. In total, cervical tissue samples from 1964 women were collected (Denmark = 502, Iceland = 389, Norway = 509, and Sweden = 564). All cervical specimens were classified based on the histological diagnosis as CIN2, CIN3 or cervical cancer. Cancers were further classified into squamous cell carcinoma (SCC), adenocarcinoma (AC), and other/cervical carcinoma not otherwise specified (NOS). In total, we excluded 85 specimens, due to inadequacy of the sample for HPV testing (n = 64), diagnosis other than CIN2, CIN3, or cervical cancer (n = 9), withdrawal of consent (n = 5), or lack of appropriate specimen identification (n = 7), thereby leaving 1879 samples for study. The age distribution of the included women in this study was comparable to the age distribution of all women with cervical high‐grade lesions and cancer in each country.^15^, ^16^

### 2.2 HPV genotyping

#### 2.2.1 Sectioning

All formalin‐fixed paraffin‐embedded blocks were sectioned according to a standardized protocol (previously verified to not result in contaminations) at a commercial, accredited laboratory, HistoCenter, Inc in Gothenburg, Sweden. Every case‐block was sectioned with a new knife, using new gloves. In between each case‐block, a blank‐block containing only paraffin was sectioned as a control for contamination. The blank‐block was sectioned first. Four 5‐µm sections were transferred to a 1.5 mL Eppendorf screw cap tube, using a new toothpick. The case‐block was mounted on the microtome and six sections, 5 µm each, were made with the same knife as the blank‐block. The first and last section were stained with hematoxylin‐eosin for rereview by a senior pathologist to assess the histology on the actual tissue, and the four 5‐µm sections in‐between were transferred to a tube in the same way as the sections from the blank‐block. After each case‐block, the knife was “removed and the microtome cleaned with DNAZap (Applied Biosystems, Waltham, MA, USA).

#### 2.2.2 DNA extraction, HPV genotyping, and Sanger sequencing

All samples, case‐blocks and blank‐blocks, were extracted with Xylene as previously described.^17^ Briefly, 1 mL Xylene per sample was incubated in 50°C for 30 minutes followed by vortexing and centrifugation 3000 *g*, for 10 minutes. The supernatant was removed with a sterile transfer pipette and the process repeated once. After the second removal of xylene, the samples were washed with pure ethanol twice and air‐dried. Dried pellets were incubated in 100 µL Digestion buffer (50 mmol/L Tris HCl, 1 mmol/L EDTA, pH 8.5) with Proteinase K (50 mg/mL) in 37°C for about 24 hours and then boiled at 100°C for 10 minutes, to inactivate Proteinase K. After extraction, case‐blocks and blank‐blocks were subjected to polymerase chain reaction (PCR) with modified general primers (MGP) targeting L1, and HPV genotyped using Luminex, as previously described.^18^, ^19^ Briefly, 44 different type‐specific beads were used, 39 for different HPV types, three for HPV type variants and two with “universal” probes. If a case was HPV negative, both the case‐block and the corresponding blank‐block were diluted 1/10 and analyzed again. If still HPV negative, a 1/50 dilution was done and the analysis repeated again. Case‐blocks and blank‐blocks were treated in the same way through the whole process.

Betaglobin real‐time PCR was performed^20^ on both blank‐blocks and case‐blocks to detect the absence and presence of human DNA. In all analyses, 1 µL extracted material was used in all PCRs with a total volume of 25 µL. For a valid result, the blank‐block had to be negative in both the betaglobin and the HPV test and the case‐block had to be positive for betaglobin.

For case‐blocks with a valid result positive for a universal probe, but not for any specific HPV type, the MGP PCR product was separated with 2% agarose gel electrophoresis. Fragments of length 170 bp were cut out and purified using QIAquick Gel Extraction Kit (Qiagen, Venlo, The Netherlands), 30 µL elution buffer. After purification, the product was sent for Sanger sequencing at Eurofins MWG Operon, Ebersberg, Germany.

### 2.3 Statistical analysis

We assessed the HPV prevalence anddistribution of HPV types in the CIN2, CIN3, and cervical cancer specimens. The prevalences are shown both among the whole study population and among HPV‐positive women. The following HPV types were defined as oncogenic: HPV16, 18, 31, 33, 35, 39, 45, 51, 52, 56, 58, 59, or probably oncogenic: HPV68, HPV types.^21^ We assessed the prevalence of HPV16 and/or 18 (HPV16/18), HPV31, 33, 45, 52, and/or 58 (HPV31/33/45/52/58), and HPV negatives in CIN2, CIN3, and cervical cancer specimens, according to the women’s age at diagnosis. In addition, among HPV16/18 positive, HPV31/33/45/52/58 positive and HPV‐negative women, we tested for trend in age using logistic regression with women’s age at diagnosis as a continuous variable. Age at diagnosis was categorized in 5‐year intervals for CIN: ≤24, 25‐29, 30‐34, 35‐39, and ≥40 years, and in 10‐year intervals for cervical cancer: ≤34, 35‐44, 45‐54, and ≥55 years. Finally, we estimated the potential preventive effect for an HPV vaccine protecting against two oncogenic types (16/18), and an HPV vaccine protecting against seven oncogenic HPV types (16/18/31/33/45/52/58), by adding the number of cases caused by these vaccine HPV types alone or in combination with other types. We also analyzed the HPV prevalence and HPV type distribution by country, but as the results for the four countries were similar, only the combined data are shown. We compared the HPV prevalence in CIN2, CIN3, and cervical cancer between the four countries, and in addition, we compared the HPV prevalence between SCC and AC by use of the chi‐squared test. All statistical analyses were performed using SAS Software version 9.4 (SAS Institute, Cary, NC, USA), with a significance level of 0.05.

## 3 RESULTS

In the present study, we included specimens from 1240 high‐grade CIN lesions (404 CIN2 and 836 CIN3) and 639 cervical cancer specimens (480 SCC, 108 AC; Table 1). The median age at diagnosis of CIN ranged from 30 years in Iceland to 34 years in Norway with an overall median age of 31 years. The median age at diagnosis for cervical cancer ranged from 41 years in Denmark to 51 years in Sweden with an overall median age of 46 years among all women. Overall, the proportion of specimens that were HPV positive was in the range 75.7%‐86.3% for CIN2; 88.3%‐94.7% for CIN3 and 82.4%‐92.2% for cervical cancer (Table 1). The proportions of HPV‐positive cancer specimens from Norway and Sweden tended to be lower than for Denmark and Iceland, but the difference between the four countries did not quite reach statistical significance (*P *= 0.079).

**Table 1:** Country‐specific characteristics of the study population (N = 1879)

	Total (N = 1879)		Denmark (n = 491)		Norway (n = 472)		Sweden (n = 537)		Iceland (n = 379)	
	n	% HPV pos.	n	% HPV pos.	n	% HPV pos.	n	% HPV pos.	n	% HPV pos.
Histological diagnosis										
CIN (total)	1240	88.7	289	87.2	285	86.7	389	90.8	277	89.5
CIN2^a^	404	82.7	100	82.0	37	75.7	182	86.3	85	78.8
CIN3^b^	836	91.6	189	90.0	248	88.3	207	94.7	192	94.3
Cervical cancer (total)^c^	639	86.4	202	88.6	187	82.4	148	84.5	102	92.2
Squamous cell carcinoma	480	89.8	163	92.0	123	86.2	120	85.8	74	97.3
Adenocarcinoma	108	72.2	22	68.2	44	72.7	19	68.4	23	78.3
Other/cervical carcinoma NOS	51	84.3	17	82.4	20	80.0	9	100.0	5	80.0
Age at diagnosis (y) [median (range)]										
CIN	31 (19‐88)		31 (20‐78)		34 (19‐88)		31 (19‐83)		30 (19‐75)	
Cervical cancer	46 (20‐98)		41 (23‐92)		48 (23‐98)		51 (22‐90)		45 (20‐91)	

CIN, cervical intraepithelial neoplasia; HPV pos., human papillomavirus positive; NOS, not otherwise specified.

a *P*‐value for the difference between countries: *P* = 0.282.

b *P*‐value for the difference between countries: *P* = 0.036.

c *P*‐value for the difference between countries: *P* = 0.079.

Table 2 presents the distribution of HPV types among women with high‐grade precancerous cervical lesions for the four countries combined. In our study, 82.7% (95% CI 79.0‐86.4) of the specimens from women with CIN2 and 91.6% (95% CI 89.7‐93.5) of the specimens from women with CIN3 were HPV positive. A few samples had only nononcogenic HPV types (CIN2: 5.2%, 95% CI 3.0‐7.4 and CIN3: 1.6%, 95% CI 0.7‐2.4). Most CIN2 and CIN3 specimens harbored only a single type of oncogenic HPV: CIN2: 63.1% (95% CI 58.4‐67.8) and CIN3: 74.4% (95% CI 71.4‐77.4). The most prevalent HPV type was HPV16 in CIN2 (35.9%, 95% CI 31.2‐40.6 among all and 43.4%, 95% CI 38.1‐48.8 among HPV positive) and CIN3 (50.2%, 95% CI 46.8‐53.6 among all and 54.8%, 95% CI 51.3‐58.4 among HPV positive). The next most common type was HPV31 (CIN2: 10.9%, 95% CI 7.8‐13.9 among all and CIN3: 12.1%, 95% CI 9.9‐14.3 among all), followed by (in decreasing order) HPV52, 51 and 33 among women with CIN2 and HPV33, 52, and 18 among women with CIN3. HPV70 was the most prevalent nononcogenic HPV type both in CIN2 and in CIN3.

**Table 2:** Distribution of HPV types among women with high‐grade cervical intraepithelial neoplasia (CIN) from four Nordic countries (n = 1240)

	CIN2			CIN3		
	n	Among total (n = 404)	Among HPV pos. (n = 334)	n	Among total (n = 836)	Among HPV pos. (n = 766)
		%	%		%	%
HPV result						
Negative	70	17.3		70	8.4	
Positive	334	82.7		767	91.6	
Oncogenic only	284	70.3	85.0	714	85.4	93.2
Nononcogenic only	21	5.2	6.3	13	1.6	1.7
Oncogenic and nononcogenic	29	7.2	8.7	39	4.7	5.1
No. of oncogenic HPV types						
1	255	63.1	76.4	622	74.4	81.2
2	40	9.9	12.0	111	13.3	14.5
3‐4	18	4.5	5.4	20	2.4	2.6
Oncogenic HPV types^a^						
16	145	35.9	43.4	420	50.2	54.8
18	26	6.4	7.8	66	7.9	8.6
31	44	10.9	13.2	101	12.1	13.2
33	28	6.9	8.4	94	11.2	12.3
35	10	2.5	3.0	20	2.4	2.6
39	16	4.0	4.8	12	1.4	1.6
45	10	2.5	3.0	35	4.2	4.6
51	31	7.7	9.3	22	2.6	2.9
52	44	10.9	13.2	67	8.0	8.7
56	13	3.2	3.9	17	2.0	2.2
58	16	4.0	4.8	24	2.9	3.1
59	4	1.0	1.2	20	2.4	2.6
68	2	0.5	0.6	7	0.8	0.9
No. of nononcogenic HPV types						
1	47	11.6	14.1	46	5.5	6.0
2‐4	3	0.7	0.9	6	0.7	0.8
Nononcogenic HPV types						
6	8	2.0	2.4	7	0.8	0.9
11	6	1.5	1.8	1	0.1	0.1
26	1	0.3	0.3	1	0.1	0.1
42	5	1.2	1.5	4	0.5	0.5
43	0	0.0	0.0	2	0.2	0.3
53	3	0.7	0.9	5	0.6	0.7
66	7	1.7	2.1	7	0.8	0.9
67	7	1.7	2.1	6	0.7	0.8
69	0	0.0	0.0	2	0.2	0.3
70	9	2.2	2.7	14	1.7	1.8
73	2	0.5	0.6	1	0.1	0.1
81	1	0.3	0.3	4	0.5	0.5
89	3	0.7	0.9	3	0.4	0.4
91	1	0.3	0.3	1	0.1	0.1

HPV pos., human papillomavirus positive.

a The sum of specific oncogenic HPV types exceeds the total number of HPV positive due to multiple infections.

The distribution of HPV types among women with cervical cancer is displayed in Table 3. Overall, HPV was detected in 86.4% (95% CI 83.7‐89.1) of cervical cancer. The HPV detection rate was higher in SCC (89.8%, 95% CI 87.1‐92.5) than in AC (72.2%, 95% CI 63.6‐80.8; *P *< 0.0001). Infection with single HPV types was the commonest among cervical cancer overall (80.9%, 95% CI 77.9‐84.0), both in SCC (83.1%, 95% CI 79.8‐86.5) and AC (72.2%, 95% CI 63.6‐80.8). HPV16 and HPV18 were the most frequent HPV types in cervical cancer overall (48.8%, 95% CI 44.9‐52.7 and 15.3%, 95% CI 12.5‐18.1, respectively). Among all SCC specimens, the prevalence of HPV16 was 53.3% (95% CI 48.9‐57.8), followed by HPV18 (9.8%, 95% CI 7.1‐12.5), HPV33, 45, and 31. HPV16 was also the most prevalent HPV type among all AC specimens (36.1%, 95% CI 26.9‐45.3), while HPV18 accounted for almost all the remaining infections within this group (30.6%, 95% CI 21.7‐39.4). The prevalence of nononcogenic HPV types was very low in cervical cancer, ranging from 0% to 0.6%.

**Table 3:** Distribution of HPV types among women with cervical cancer from four Nordic countries (n = 639)

	Cervical cancer (total)^a^			Squamous cell carcinoma			Adenocarcinoma		
	n	Among total (n = 639)	Among HPV pos. (n = 552)	n	Among total (n = 480)	Among HPV pos. (n = 431)	n	Among total (n = 108)	Among HPV pos. (n = 78)
		%	%		%	%		%	%
HPV result									
Negative	87	13.6		49	10.2		30	27.8	
Positive^b^	552	86.4		431	89.8		78	72.2	
Oncogenic only	535	83.7	96.9	416	86.7	96.5	76	70.4	97.4
Nononcogenic only	11	1.7	2.0	11	2.3	2.6	0	0.0	0.0
Oncogenic and nononcogenic	6	0.9	1.1	4	0.8	0.9	2	1.9	2.6
No. of oncogenic HPV types									
1	517	80.9	93.7	399	83.1	92.6	78	72.2	100.0
2‐3	24	3.8	4.4	21	4.4	4.8	0	0.0	0.0
Oncogenic HPV types^c^									
16	312	48.8	56.5	256	53.3	59.4	39	36.1	50.0
18	98	15.3	17.8	47	9.8	10.9	33	30.6	42.3
31	21	3.3	3.8	21	4.4	4.9	0	0.0	0.0
33	35	5.5	6.3	33	6.9	7.7	1	0.9	1.3
35	10	1.6	1.8	8	1.7	1.9	0	0.0	0.0
39	15	2.4	2.7	15	3.1	3.5	0	0.0	0.0
45	41	6.4	7.4	31	6.5	7.2	5	4.6	6.4
51	0	0.0	0.0	0	0.0	0.0	0	0.0	0.0
52	10	1.6	1.8	9	1.9	2.1	0	0.0	0.0
56	6	0.9	1.1	5	1.0	1.2	0	0.0	0.0
58	4	0.6	0.7	4	0.8	0.9	0	0.0	0.0
59	14	2.2	2.5	13	2.7	3.0	0	0.0	0.0
68	0	0.0	0.0	0	0.0	0.0	0	0.0	0.0
No. of nononcogenic HPV types									
1	17	2.7	3.1	15	3.1	3.5	2	1.9	2.6
Nononcogenic HPV types									
6	3	0.5	0.5	2	0.4	0.5	1	0.9	1.3
11	1	0.2	0.2	1	0.2	0.2	0	0.0	0.0
26	0	0.0	0.0	0	0.0	0.0	0	0.0	0.0
42	1	0.2	0.2	1	0.2	0.2	0	0.0	0.0
43	0	0.0	0.0	0	0.0	0.0	0	0.0	0.0
53	0	0.0	0.0	0	0.0	0.0	0	0.0	0.0
66	3	0.5	0.5	3	0.6	0.7	0	0.0	0.0
67	3	0.5	0.5	2	0.4	0.5	1	0.9	1.3
69	0	0.0	0.0	0	0.0	0.0	0	0.0	0.0
70	4	0.6	0.7	4	0.8	0.9	0	0.0	0.0
73	1	0.2	0.2	1	0.2	0.2	0	0.0	0.0
81	1	0.2	0.2	1	0.2	0.2	0	0.0	0.0
89	0	0.0	0.0	0	0.0	0.0	0	0.0	0.0
91	0	0.0	0.0	0	0.0	0.0	0	0.0	0.0

HPV pos., human papillomavirus positive.

a Cervical cancer (total) includes squamous cell carcinoma, adenocarcinoma, and other/cervical carcinoma not otherwise specified.

b *P*‐value for the difference between SCC and AC: *P* < 0.0001.

c The sum of specific oncogenic HPV types exceeds the total number of HPV positive due to multiple infections.

**Figure 1:**
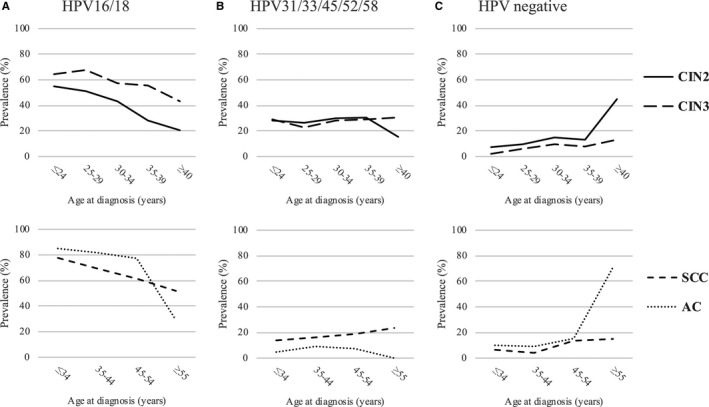
Prevalence of HPV16/18 positive (A), HPV31/33/45/52/58 positive (B) and HPV negative (C) in relation to woman’s age at diagnosis among Nordic women with CIN2, CIN3, squamous cell carcinoma or adenocarcinoma. AC, adenocarcinoma; CIN, cervical intraepithelial neoplasia; HPV, human papillomavirus; SCC, squamous cell carcinoma

Figure 1 shows the proportion of specimens that were HPV16/18 positive, HPV31/33/45/52/58 positive or HPV negative in relation to the women’s age at diagnosis. Among women with CIN2 and CIN3, the prevalence of HPV16/18 decreased with increasing age at diagnosis (*P *< 0.0001 for both). In contrast, the prevalence of HPV31/33/45/52/58 remained relatively constant in all age groups, and the prevalence of HPV‐negative samples increased with increasing age (*P* < 0.0001 for both). Among the women with SCC and AC, the prevalence of HPV16/18 decreased with increasing age at diagnosis (*P *< 0.0001 for both), whereas the prevalence of HPV31/33/45/52/58 increased slightly with increasing age among women with SSC (*P* = 0.055). The age pattern in the prevalence of HPV‐negative samples showed an increase with increasing age at diagnosis (SCC: *P* = 0.0003; AC: *P* < 0.0001).

**Figure 2:**
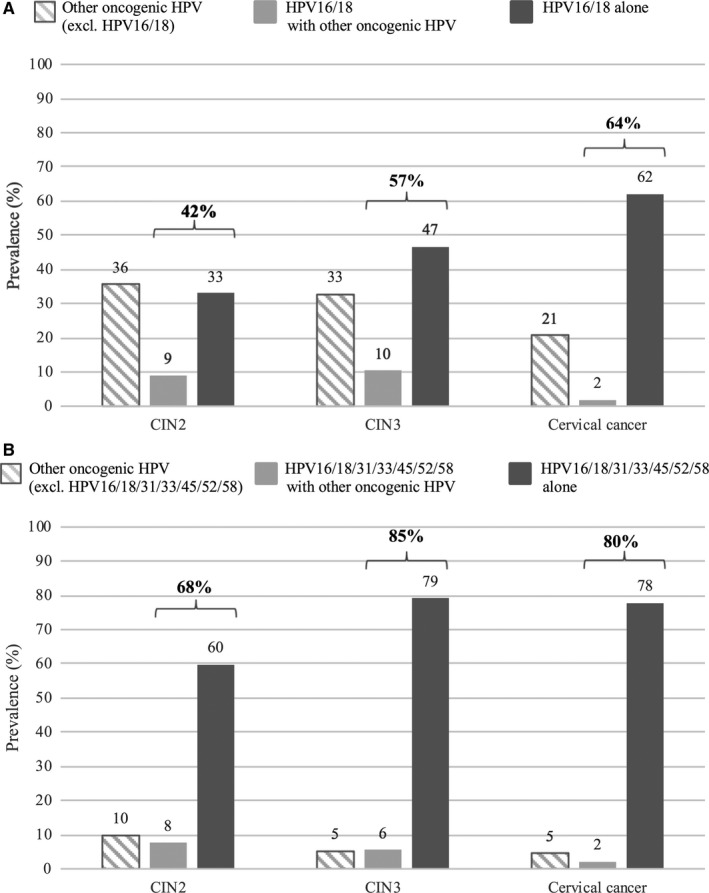
Estimated potential preventive effect of HPV 16/18 vaccine (A) and HPV 16/18/31/33/45/52/58 vaccine (B) among Nordic women with CIN2, CIN3 and cervical cancer. CIN, cervical intraepithelial neoplasia; HPV, human papillomavirus

Figure 2 shows the potential preventive effect of HPV vaccines protecting against two oncogenic HPV types (16/18) or seven oncogenic HPV types (16/18/31/33/45/52/58). In total, 42% (95% CI 37.0‐46.7), 57% (95% CI 53.8‐60.5), and 64% (95% CI 60.3‐67.7) of CIN2, CIN3, and cervical cancer, respectively, could be prevented, if women were vaccinated with an HPV vaccine preventing two oncogenic HPV types (16/18), assuming that HPV16/18 caused the lesion, if present (corresponding estimates among HPV positive: CIN2: 51%, 95% CI 45.2‐56.0, CIN3: 62%, 95% CI 59.0‐65.8, and cervical cancer: 74%, 95% CI 70.4‐77.8). Lesions positive for HPV16/18, but no other oncogenic HPV, accounted for 33% (95% CI 28.3‐37.5), 47% (95% CI 43.4‐50.2), and 62% (95% CI 58.4‐65.9) of CIN2, CIN3, and cervical cancer, respectively (corresponding estimates among HPV positive: CIN2: 40%, 95% CI 34.5‐45.1, CIN3: 51%, 95% CI 47.5‐54.6 and cervical cancer: 72%, 95% CI 68.2‐75.7). The preventive potential increased to 68% (95% CI 63.0‐72.2) for CIN2, 85% (95% CI 82.4‐87.2) for CIN3, and 80% (95% CI 77.0‐83.2) for cervical cancer if five additional oncogenic HPV types (31/33/45/52/58) were included in the vaccine (corresponding estimates among HPV positive: CIN2: 82%, 95% CI 77.6‐85.9, CIN3: 93%, 95% CI 90.7‐94.4 and cervical cancer: 93%, 95% CI 90.6‐94.9).

## 4 DISCUSSION

In this large multicountry study on cervical specimens obtained in the period 2004‐2006 from almost 2000 women with high‐grade precancerous cervical lesions or cervical cancer, we found that HPV16 and 31 were the most common types in CIN2 and CIN3, while HPV16 and 18 were the most common types in cervical cancer, with HPV18 being far more common in AC than in SCC. In CIN2 and CIN3 as well as cancer specimens, the prevalence of HPV‐negative samples increased with increasing age at diagnosis, while the prevalence of HPV types 16/18 was found to decrease. The preventive potential of HPV16/18 vaccination was 57% and 64% in CIN3 and cervical cancer, respectively, while inclusion of HPV31/33/45/52/58 in a vaccine increased the protection to more than 80% in both the groups.

Other studies also report HPV16 as the most prevalent type in women with CIN2, CIN3, and cervical cancer, followed by HPV31 in CIN2 and CIN3 and HPV18 in cancer.^4^, ^22^, ^23^ The majority of the oncogenic HPV types detected in our study were present as single type HPV infections, ranging from around 63% in CIN2 to 81% in cervical cancer. In the relatively few samples with multiple infections, it can be difficult to determine which type caused the lesion.^24^ Our findings comparing AC and SCC also confirm that the contribution of HPV18 is substantially higher in AC than in SCC.^4^

With increasing age at diagnosis of the women, the specimens were less likely to harbor HPV16/18. A decrease in the prevalence of HPV16/18 with respect to woman’s age at diagnosis has been reported in previous studies.^13^, ^14^, ^25^ HPV16 possesses a unique carcinogenic potential compared to other HPV types, and has a higher probability of persistence, and an overall increasing prevalence with increasing severity of the cervical lesion.^26^, ^27^ The transition from normal epithelium to CIN and cervical cancer appears to be faster in HPV16/18‐positive women than in women positive for other HPV types, which correlates well with reports of a lower mean age at diagnosis in women with HPV16/18‐positive CIN and cervical cancer compared to those positive for non‐HPV16/18 HPV types.^11^, ^28^, ^29^ Furthermore, CIN lesions with HPV16 infection found in screening programs may be larger and easier for colposcopists to identify and remove compared with non‐HPV16 positive lesions, which could lead to a lower prevalence of HPV16‐related cancers among older women.^30^ Finally, our results may in theory be explained by a cohort effect. In contrast, the prevalence of HPV31/33/45/52/58 displayed a more stable prevalence across age groups, which is also in line with previous findings.^5^

Infection with HPV is recognized as a necessary cause of cervical cancer, so our finding of around 10% HPV‐negative specimens in women with cervical cancer is relatively high, although consistent with several other studies.^5^, ^12^, ^13^, ^22^ We present the distribution of HPV types among the whole study population, and among HPV‐positive women. In our study, the cancer specimens from Norway and Sweden had a higher proportion of HPV negative than the cancer samples from Denmark and Iceland, although this difference was not statistically significant. The median age at diagnosis also tended to be higher in women with cervical cancer from Norway and Sweden than from Denmark and Iceland. HPV negativity can be due to technical limitations, limited quality of biopsy specimens^31^ or integration of HPV into the host genome causing loss of the L1 sequences otherwise targeted by the MGP primer.^32^ Despite the wide range of HPV types tested for in this study, theoretically some of the HPV‐negative samples may be infected with other HPV types not detectable by this PCR method. As also reported by others,^25^, ^32^ women with AC were more likely to be tested HPV negative (27.8%) than women with SCC (10.2%). This finding may be associated with non‐HPV‐related endometrial cancer in the lower part of the uterine body being misclassified as cervical AC.^33^ Finally, a recent European multicenter study found extensive variation in the HPV prevalence (0%‐90%) among different histological subgroups of cervical AC, suggesting that a fraction of AC may actually not be HPV related.^29^ In our study, the number of AC samples was limited and AC subgroups were not assessed.

A small number of CIN3 and cervical cancer cases harbored nononcogenic HPV types only, as reported in other studies.^4^, ^32^ This finding is likely due to coinfection with an undetected oncogenic HPV type.

Since the introduction of HPV vaccination programs, ecological studies have reported declines of genital warts and CIN of different grades.^7^, ^34^ Similar results have been found by recent nationwide cohort studies with information on individual HPV vaccination status. A Danish study compared women vaccinated with at least one dose of an HPV16/18 vaccine with unvaccinated women and reported a 73% reduction in risk of CIN2/3, and 80% of CIN3 alone, for the youngest birth cohort (born 1993‐1994), which also had the highest HPV vaccination coverage.^9^ A similar study among Swedish women found a 75% reduction in risk of CIN2+, and 84% of CIN3+, when comparing fully vaccinated (three doses of an HPV16/18 vaccine, vaccination initiated before age 17) women with unvaccinated women.^8^ Kjaer et al^10^ reported HPV results from more than 40 000 Danish women in a pre‐HPV vaccination period and found a potential preventive effect of HPV16/18 vaccination of 74% in cervical cancer (CIN2: 37%; CIN3: 66%), while an HPV vaccine targeting seven oncogenic HPV types (HPV16/18/31/33/45/52/58) was predicted to prevent 89% of cervical cancer (CIN2: 80%; CIN3: 91%). These estimated preventive effects are slightly greater than the effects found among the study population in the present study, but similar to our results among HPV‐positive women and may therefore be explained by the generally higher prevalence of HPV‐positive samples in all histological categories in Kjær et al^10^ than in the present study. It should be noted that the HPV testing in that study was done on cytology samples, whereas in the present study HPV testing was performed on histological specimens.

In the present study, we found a substantial effect of including five additional oncogenic HPV types compared to an HPV16/18 vaccine among women with high‐grade precancerous cervical lesions, where the potential prevention increased from 42% to 68% in CIN2 and from 57% to 85% in CIN3. This illustrates the high prevalence of especially HPV31, 33, and 52 in CIN2 and CIN3. Furthermore, this underlines the potential impact of a vaccine including HPV16/18/31/33/45/52/58 or a vaccine with substantial cross‐protection against these types on the future incidence of high‐grade CIN among Nordic women. When comparing the effect of an HPV16/18 vaccine to an HPV16/18/31/33/45/52/58 vaccine on cervical cancer, the estimated prevention increased from 64% to 80% (among HPV positive: 74%‐93%), corresponding to results from a recent population‐based US study and a meta‐analysis of two worldwide HPV distribution studies.^5^, ^31^ Based on recent incidence data on cervical cancer from the Nordic region, around 1200 women are at present diagnosed with cervical cancer in Denmark, Iceland, Norway, and Sweden combined every year.^16^ Implementation of an HPV16/18 vaccine would prevent approximately 750 cervical cancers per year, while the use of an HPV16/18/31/33/45/52/58 vaccine would increase this estimated preventive effect to around 940 cervical cancers. Moreover, since all of the oncogenic HPV types we detected in the AC specimens are included in the HPV16/18/31/33/45/52/58 vaccine, we estimate a potential 100% reduction in HPV‐positive AC, under the assumption of full vaccination coverage and long‐term effectiveness.

Strengths of our study include the population‐based design, the large sample size, and inclusion of women from four Nordic countries with a similar demographic composition, thus making our results generalizable to the Nordic female population. Our HPV results were from histologically confirmed diagnostic categories (CIN2, CIN3, and cervical cancer), which increases the likelihood of a true causal association. HPV genotyping was performed by use of a highly sensitive PCR method at a central reference laboratory to ensure standardized testing. In addition, we tested for a wide spectrum of HPV types. Previous studies have reported results for CIN or cervical cancer overall, or in some cases for combined CIN2+ or CIN3+ groups, whereas we provide results for CIN2 and CIN3 separately, as well as histological subgroups (SCC, AC) of cervical cancer, to fully report the differences in specific genotypes between groups.^32^, ^35^ Furthermore, we show age‐stratified results of HPV16/18 and HPV31/33/45/52/58 prevalence, which is important because the HPV16/18 prevalence has been shown to differ between age groups.^23^, ^28^, ^32^ Our study also has limitations. Despite the high sensitivity of the PCR method, some HPV infections may have not been detected. On the other hand, very sensitive HPV testing may detect latent or transient HPV infections, which are not necessarily causal.

In conclusion, we present here a baseline assessment of the overall and type‐specific HPV prevalence and age‐specific distribution in both CIN2 and CIN3 as well as cervical cancer among women from four Nordic countries before implementation of HPV vaccination. These results form the basis for future monitoring of the effect of HPV vaccination. In addition, they contribute with estimates of the potential preventive impact of both HPV16/18 and HPV16/18/31/33/45/52/58 vaccination in the Nordic countries.

## CONFLICT OF INTEREST

SKK received lecture fee from Merck and Sanofi Pasteur MSD, scientific advisory board fee from Merck, and unrestricted research grants through her institution from Merck. JD reports having received research grants to his institution for the funding of the study. CM received lecture fees and travel grants from Sanofi Pasteur MSD. CL and MH report that their institution received a grant from Merck. KLL is a full‐time employee of Merck & Co. Inc and owns stocks and options of Merck. MN received research grants from MSD Norway/Merck through the affiliating institute. CDC, SG, LT, KS, and BTH report no conflicts of interest.
